# Vital members in the gut microbiotas altered by two probiotic *Bifidobacterium* strains against liver damage in rats

**DOI:** 10.1186/s12866-020-01827-2

**Published:** 2020-06-05

**Authors:** Hua Zha, Dai-Qiong Fang, Aimee van der Reis, Kevin Chang, Li-Ya Yang, Jiao-Jiao Xie, Ding Shi, Qiao-Mai Xu, Ya-Ting Li, Lan-Juan Li

**Affiliations:** 1 State Key Laboratory for Diagnosis and Treatment of Infectious Diseases, Collaborative Innovation Center for Diagnosis and Treatment of Infectious Diseases, National Clinical Research Center for Infectious Diseases, The First Affiliated Hospital, College of Medicine, Zhejiang University, 79 Qingchun Road, Hangzhou, 310000 China; 2 School of Biological Sciences, The University of Auckland, Auckland, New Zealand; 3 Institute of Marine Science, The University of Auckland, Auckland, New Zealand; 4 Department of Statistics, The University of Auckland, Auckland, New Zealand

**Keywords:** Gut microbiota, Probiotic bacteria, Liver injury, Protective effect, Illumina sequencing, Microbial dysbiosis status

## Abstract

**Background:**

Probiotics are effective to rectify the imbalanced gut microbiota in the diseased cohorts. Two *Bifidobacterium* strains (LI09 and LI10) were found to alleviate D-galactosamine-induced liver damage (LD) in rats in our previous work. A series of bioinformatic and statistical analyses were performed to determine the vital bacteria in the gut microbiotas altered by the LI09 or LI10 in rats.

**Results:**

Two groups of representative phylotypes could distinguish the gut microbiotas of LI09 or LI10 groups from the other groups. Among them, OTU170_*Porphyromonadaceae* acted as a gatekeeper in LI09 group, while OTU12_*Bacteroides* was determined with multiple correlations in the gut network of LI10 group. Multiple reduced OTUs associated with LC and increased OTUs associated with health were determined in LI09 or LI10 groups, among which, increased OTU51_*Barnesiella* and reduced OTU99_*Barnesiella* could be associated with the protective effects of both the two probiotics. The gut microbiotas in LI09, LI10 and positive control groups were clustered into three clusters, i.e., Cluster_1_Microbiota, Cluster_2_Microbiota and Cluster_3_Microbiota, by Partition Around Medoids clustering analysis. Cluster_2_Microbiota was determined at least dysbiotic status due to its greatest LD dysbiosis ratio, lowest levels of liver function variables and plasma cytokines compared with the two other clustered microbiotas, suggesting the treated rats in Cluster_2 were at better health status.

**Conclusion:**

Our findings suggest that OTU170_*Porphyromonadaceae* and OTU12_*Bacteroides* are vital in the gut microbiotas altered by LI09 and LI10. Characteristics of the LD cohorts treated by LI09 or LI10 at different gut microbial colonization states could help monitor the cohorts’ health status.

## Background

Liver damage (LD) can result in significant morbidity or mortality in human from different countries [1, 2]. Multiple factors are associated with LD [2–5], among which, altered gut microbiota is attracting increasing scientific interests [6, 7]. The beneficial effects of probiotics against diseases have been well reported [8–12], and different probiotics were proved capable of rectifying an imbalanced gut microbiome in LD cohorts or animal models [13–16].

Probiotics have been reported with diverse but valuable functions associated with the protective effects against LD [14, 15]. Administrations of *Lactobacilli acidophilus* NM1, *Lactobacillus rhamnosus* ATCC 53103, or *L. rhamnosus* DSM 6594+ *Lactobacillus plantarum* DSM 9843 could decrease bacterial translocation in the rat models compared with the LD positive control group [17]. *L. rhamnosus* (Gorbach-Goldin) could help preserve the normal barrier function of a rat model against alcohol-induced LD [14]. *L. plantarum* (DSM 15313) and *Bifidobacterium infantis* (DSM 15159) were able to reduce alanine aminotransferase and increase liver glutathione levels in endotoxin- and D-galactosamine-induced LD [18].

In our previous study, two probiotic *Bifidobacterium* strains, i.e., *Bifidobacterium pseudocatenulatum* LI09 and *Bifidobacterium catenulatum* LI10, were capable of alleviating D-galactosamine-induced LD via modifying the gut microbiota and improving the liver function in rats [15]. The present study is designed to further determine 1) the vital members in the gut microbiotas of LD rats altered by LI09 or LI10, 2) whether the characteristics of LI09 or LI10 treated LD cohorts at different gut microbial colonization states could help monitor the health status of the cohorts.

## Results

### Differences between the gut microbiotas of the seven groups

None of the rats died after the induction of LD [15], and all the survived rats were used for all the subsequent analyses. There is a significant difference among the gut microbiotas of the seven groups based on the Permutation analysis of variance (PERMANOVA) results (*R*^*2*^ = 0.176, *P* < 0.001). Similarity percentage (SIMPER) analysis determined a range of dissimilarities (60.91 to 68.27%) between the gut microbiotas of five probiotics groups (*n* = 9 per group) and those of positive control (PC, *n* = 8) or negative control (NC, *n* = 6) groups. The dissimilarities between the gut microbiotas in the five probiotics groups and PC group were similar to those between five probiotics groups and NC group (t-test, *P* > 0.70).

### Representative operational taxonomic units (OTUs) and functional metabolites associated with the gut microbiotas altered by LI09 or LI10

**Fig. 1:**
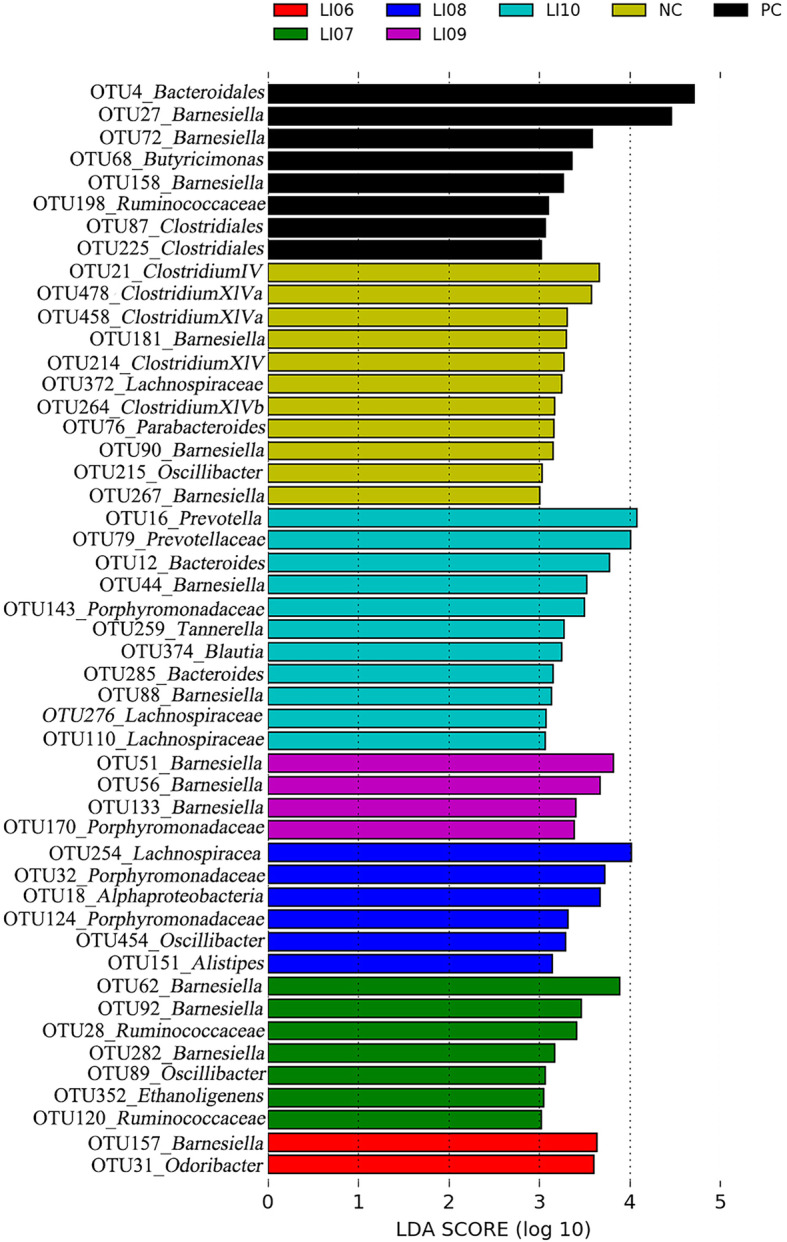
Representative OTUs belonging to each of seven groups identified by Linear Discriminant Analysis Effect Size (LEfSe)

Linear Discriminant Analysis (LDA) Effect Size (LEfSe) identified multiple representative OTUs to the gut microbiotas altered by LI09 or LI10 (Fig. 1). Three OTUs (i.e., OTUs 51, 56 and 133) from *Barnesiella* and OTU170 from *Porphyromonadaceae* were determined as representative phylotypes to LI09 group (Fig. 1). Eleven OTUs assigned to eight different taxa, i.e., *Bacteroides*, *Barnesiella*, *Blautia*, *Lachnospiraceae*, *Porphyromonadaceae*, *Prevotella*, *Prevotellaceae* and *Tannerella*, were determined as representative phylotypes to LI10 group (Fig. 1).

**Fig. 2:**
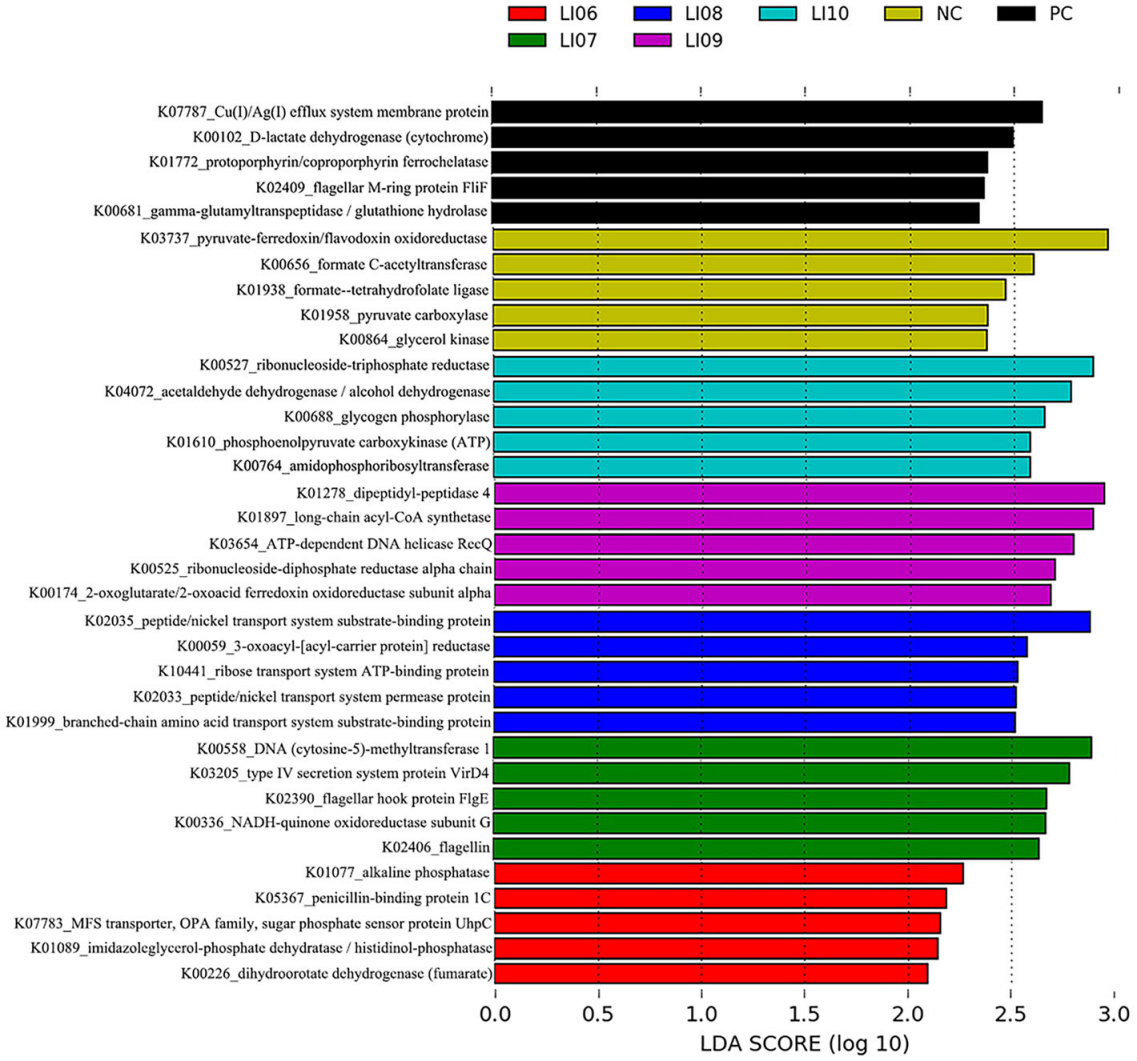
Top five functional metabolites associated with each of the seven groups determined by LEfSe

A range of functional metabolites were determined being associated with each of the seven groups, and the top five OTUs with the largest LDA scores in each group were demonstrated (Fig. 2). K01278 - dipeptidyl-peptidase 4 and K00527 - ribonucleoside-triphosphate reductase were the functional metabolites most associated with LI09 and LI10 groups, respectively.

### Bacterial networks and gatekeepers in the gut microbiotas altered by LI09 and LI10

Seven bacterial networks belonging to the seven experimental groups were determined by Co-occurrence Network inference (CoNet) analysis (Figure S1), each with distinct top 10 OTUs with most correlations (Table S1). OTU173_*Porphyromonadaceae* was determined with many correlations in the bacterial networks of both LI09 and NC groups (Table S1).

Network fragmentation analysis determined a range of one to four OTUs as gatekeepers in LI06, LI07, LI08, NC and PC groups (Table S2). No OTU associated with LI10 group acted as gatekeeper to the bacterial network of LI10 group. By contrast, nine OTUs were identified as gatekeepers to the bacterial network in LI09 group (all *P* < 0.04). One representative OTU to LI09 group, i.e., OTU170_*Porphyromonadaceae*, was also determined as a gatekeeper in LI09 group.

### Changes of OTUs associated with LD or health in LI09 and LI10 groups

**Fig. 3:**
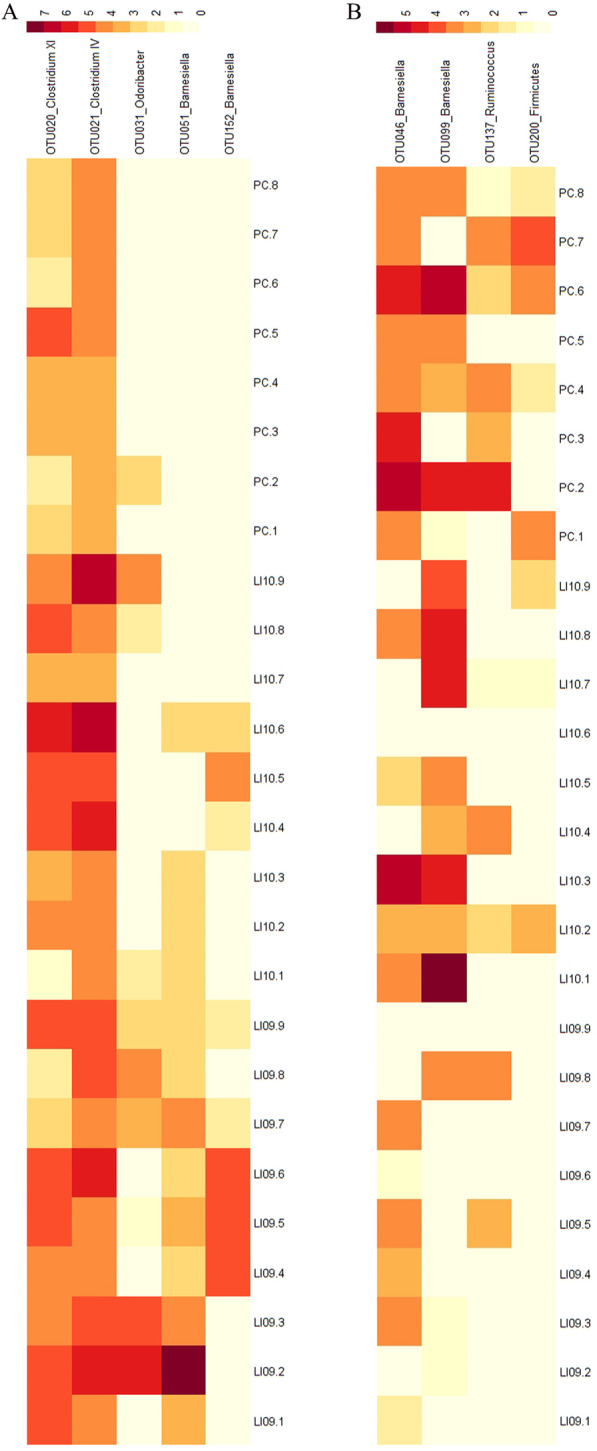
The OTUs associated with (**a**) negative control (NC) or (**b**) positive control (PC) with significant different abundances between LI09 and PC groups, or between LI10 and PC groups

LEfSe results revealed that a total of 25 OTUs associated with health, while 14 OTUs were associated with LD (Figure S2). Seven out of the 25 OTUs associated with health were determined with different abundances among LI09, LI10 and PC groups (Kruskal-Wallis test, all *P* < 0.05). Four (out of the seven) OTUs, i.e., OTU20_*Clostridium* XI, OTU21_*Clostridium* IV, OTU31_*Odoribacter*, OTU51_*Barnesiella* and OTU152_*Barnesiella*, were less abundant in PC group than LI10 group (Mann-Whitney test, all *P* < 0.05) (Fig. 3a). By contrast, only OTU51_*Barnesiella* had different abundances between LI09 and PC groups (i.e., more abundant in LI09 group) (Mann-Whitney test, *P* < 0.05) (Fig. 3a). The two remaining OTUs, i.e., OTU71_*Ruminococcaceae* and OTU110_*Lachnospiraceae*, were determined with similar abundances between LI09 and PC groups, and between LI10 and PC groups.

Similarly, six out of 14 OTUs associated with LD had different abundances between LI09, LI10 and PC groups (Kruskal-Wallis test, *P* < 0.05). Four (out of the six) OTUs, i.e., OTU99_*Barnesiella*, OTU137_*Ruminococcus*, OTU200_*Firmicutes* and OTU46_*Barnesiella*, were less abundant in LI10 group than PC group (Mann-Whitney test, all *P* < 0.05), while only OTU99_*Barnesiella* had different abundances between LI09 and PC groups (i.e., less abundant in LI09 group) (Mann-Whitney test, *P* < 0.05) (Fig. 3b). The two remaining OTUs, i.e., OTU113_*Lachnospiraceae* and OTU158_*Barnesiella*, were determined with similar abundances between LI09 and PC groups, and between LI10 and PC groups.

### Clustering of the gut microbiotas from LI09, LI10 and PC groups

**Fig. 4:**
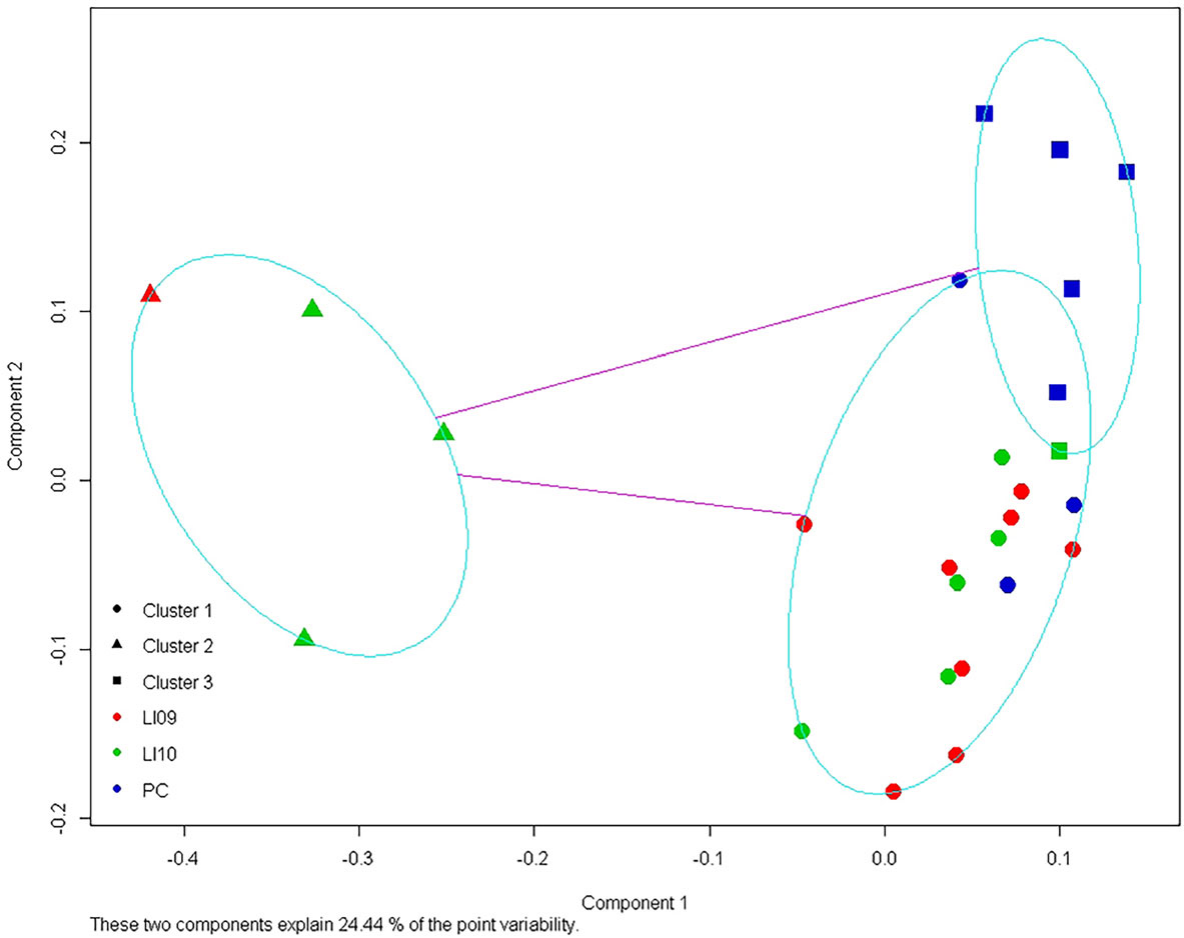
The gut microbiotas in LI09, LI10 and PC groups were clustered into three clusters by Partition around medoids (PAM) analysis

The average silhouette method was used to determine the optimal numbers of clusters for the gut microbiotas from LI09, LI10 and PC groups [19]. Two, three and six were identified with higher scores compared with other cluster numbers trialled based on the silhouette analysis results (Figure S3). Three clusters could separate PC samples from LI09 and LI10 groups well compared with two and six clusters. Therefore, the gut microbiotas in LI09, LI10 and PC groups were clustered into three clusters by Partition Around Medoids (PAM) clustering analysis (Fig. 4), each with distinct microbiotas (Table S3).

### Comparisons between the three clustered gut microbiotas

There is a significant difference among the three clustered gut microbiotas according to the PERMANOVA results (*R*^*2*^ = 0.244, *P* < 0.001). The richness (observed species) were similar between the three clustered microbiotas (one-way ANOVA, *P* > 0.35). There were significant differences in the diversity (Shannon index) and evenness (Pielou index) between the three clustered microbiotas (one-way ANOVA, all *P* < 0.02). The evenness was significantly greater in Cluster_2_Microbiota than those of Cluster_1_Microbiota or Cluster_3_Microbiota (t-test, all *P* < 0.03), while the diversity was highest in Cluster_2_Microbiota among the three other clustered microbiotas (Table S4).

There was a significant difference in LD dysbiosis ratios (LDDRs) between LI09, LI10 and PC groups (one-way ANOVA, *P* = 0.001). LDDR was greater in Cluster_2_Microbiota (8.59 ± 5.09 S.E.) than those of Cluster_1_Microbiota (0.8 ± 0.22) or Cluster_3_Microbiota (0.22 ± 0.06) (t-test, all *P* < 0.01), while the ratios were similar between Cluster_1_Microbiota and Cluster_3_Microbiota (t-test, *P* > 0.22).

### Comparisons of liver function variables and plasma cytokines in the three clustered cohorts

There were significant differences in the majority of liver function variables between the three clustered cohorts with the three clustered gut microbiotas, including alanine aminotransferase (ALT), aspartate aminotransferase (AST), glutamyltransferase (GGT), glycylproline dipeptidyl aminopeptidase (GPDA), total bile acid (TBA) and total bilirubin (TB) (one-way ANOVA, all *P* < 0.005). The six liver function variables were lower in Cluster_2 compared with Cluster_1 or Cluster_3 (t-test, all *P* < 0.01) (Table 1). Two out of six liver function variables, i.e., ALT and AST, were lower in Cluster_1 than Cluster_3 (t-test, all *P* < 0.01) (Table 1).

**Table 1:** The comparisons of liver function variables in the three clustered cohorts in LI09, LI10 and PC groups

Variable	Cluster_1	Cluster_2	Cluster_3
ALB (g/L)	36 ± 1 ^a^	39 ± 1 ^a^	35 ± 2 ^a^
ALT (U/L)	5628 ± 610 ^a^	80.0 ± 1.0 ^b^	10,393 ± 876 ^c^
AST (U/L)	7670 ± 854 ^a^	230 ± 25 ^b^	12,367 ± 932 ^c^
TBA (μmol/L)	351 ± 27 ^a^	63 ± 17 ^b^	448 ± 26 ^a^
TB (μmol/L)	33 ± 13 ^a^	2 ± 0.4 ^b^	53 ± 30 ^a^
GGT (U/L)	17 ± 2 ^a^	0.5 ± 0.3 ^b^	21 ± 4 ^a^
GPDA (U/L)	331 ± 26 ^a^	66 ± 4 ^b^	427 ± 24 ^a^

Note: Cluster_1 to 3 represented the three clusters of cohorts in LI09, LI10 and PC groups identified by partitioning around medoids clustering algorithm based on their intestinal bacterial compositions (see Fig. 4), e.g., Cluster_1 - a mix of rats from all the three groups, Cluster_2 - a mix of rats from LI09 and LI10 groups, Cluster_3 - a mix of remaining rats from LI10 and PC groups. Results were represented in Mean ± S.E., and the groups with different alphabets represented significant difference between the clustered cohorts determined by t-tests

Similarly, all the nine plasma cytokines were different between the three clustered cohorts (one-way ANOVA, all *P* < 0.02). Cluster_2 had overall lowest plasma cytokines compared with Cluster_1 and Cluster_3 (t-test, all *P* < 0.04) (Table 2). Three out of nine plasma cytokines, i.e., MIP-1α, MIP-3α and Interleukin (IL)-6, were lower in Cluster_1 than Cluster_3 (t-test, all *P* < 0.04) (Table 2).

**Table 2:** The comparisons of cytokines in the three clustered cohorts in LI09, LI10 and PC groups

Cytokines	Cluster_1	Cluster_2	Cluster_3
M-CSF (pg/ml)	496 ± 31 ^a^	301 ± 37 ^b^	604 ± 40 ^a^
TNF-α (pg/ml)	122 ± 10 ^a^	52 ± 9 ^a^	111 ± 16 ^a^
IL-5 (pg/ml)	556 ± 54 ^a^	277 ± 27 ^b^	569 ± 76 ^a^
IL-10 (pg/ml)	432 ± 32 ^a^	195 ± 38 ^b^	653 ± 124 ^a^
MIP-1α (pg/ml)	99 ± 13 ^a^	19 ± 1 ^b^	199 ± 35 ^c^
MIP-3α (pg/ml)	208 ± 22 ^a^	78 ± 9 ^b^	416 ± 81 ^c^
MCP-1 (pg/ml)	8792 ± 2169 ^a^	1054 ± 89 ^b^	10,522 ± 1660 ^a^
IL-1β (pg/ml)	122 ± 28 ^a^	31 ± 5 ^b^	82 ± 6 ^a^
IL-6 (pg/ml)	131 ± 16 ^a^	83 ± 38 ^a^	683 ± 350 ^b^

Note: Cluster_1 to 3 represented the three clusters of cohorts in LI09, LI10 and PC groups identified by partitioning around medoids clustering algorithm based on their intestinal bacterial communities (see Fig. 4). Results were represented in Mean ± S.E., and the groups with different alphabets represented significant difference between the clustered cohorts determined by t-tests

### Associations of representative OTUs with the variables in each of the three clustered microbiotas

**Fig. 5:**
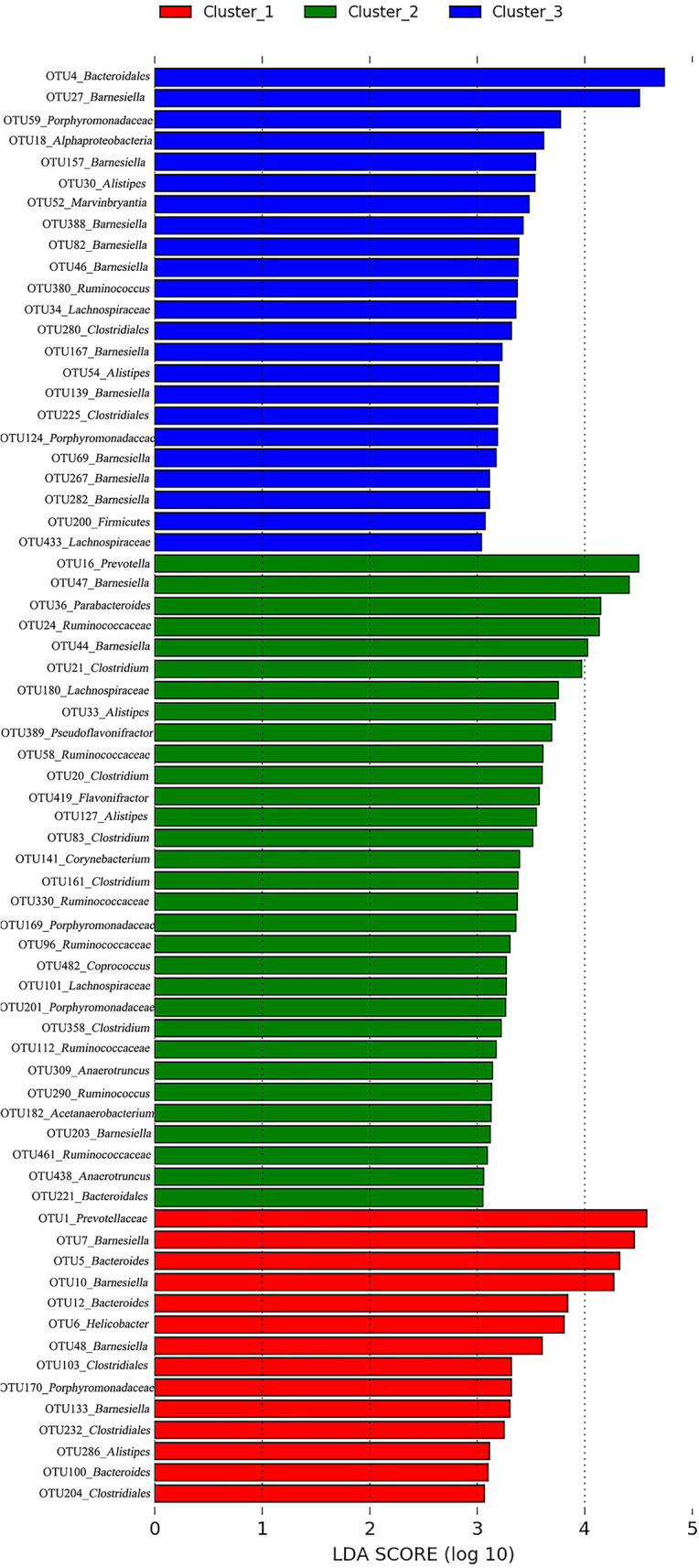
Representative OTUs belonging to each of the three clustered microbiotas of LI09, LI10 and positive control groups identified by LEfSe

Three groups of representative OTUs associated with the three clustered gut microbiotas were determined by a LEfSe analysis (Fig. 5).

**Fig. 6:**
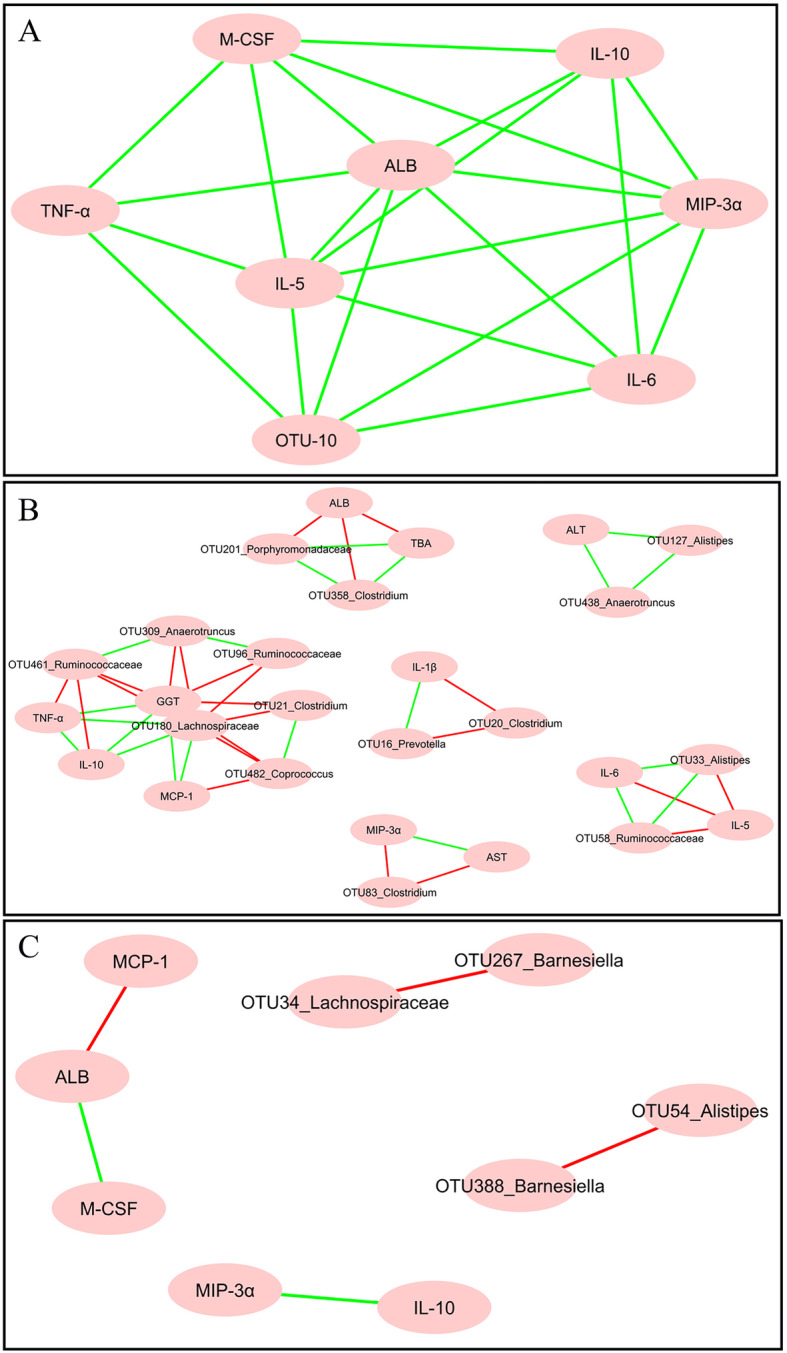
Associations of representative OTUs in the three clustered microbiotas, with liver function variables and plasma cytokines, i.e., (**a**) Cluster_1, (**b**) Cluster_2 and (**c**) Cluster_3. Note: green labelled lines represented positive correlation, and red labelled lines represented negative correlation

Only one representative OTU in Cluster_1, i.e., OTU10_*Barnesiella*, was positively associated with albumin (ALB), IL-5, IL-6, TNF-α and MIP-3α. No OTU was associated with any measured liver function variable or plasma cytokine in Cluster_3. By contrast, a group of 15 OTUs were associated with 12 liver function variables and plasma cytokines in Cluster_2 (Fig. 6). In Cluster_2, OTU180_*Lachnospiraceae* was negatively associated with five other representative OTUs (i.e., OTU21_*Clostridium*, OTU96_*Ruminococcaceae*, OTU309_*Anaerotruncus*, OTU461_*Ruminococcaceae* and OTU482_*Coprococcus*) but positively associated with four variables (Fig. 6), while the five representative OTUs were negatively associated with some of the four variables (Fig. 6).

## Discussion

Previous research suggested *B. pseudocatenulatum* LI09 and *B. catenulatum* LI10 could alleviate D-galactosamine-induced LD by improving liver function variables and plasma cytokines, as well as modifying the gut microbiota [15]. In the current study, a series of bioinformatic and statistical analyses were applied to further explore the vital members in the gut microbiotas altered by LI09 and LI10 against LD, as well as to determine the characteristics of LI09 or LI10 treated LD rats which were at different gut microbial colonization states.

The gut microbiotas in the five probiotics groups had relatively high dissimilarities with those in PC or NC groups according to SIMPER results, and the microbiotas in the five probiotics groups were not similar to the microbiotas in either PC or NC group. The top 10 phylotypes with most correlations in the bacterial networks in the five probiotics groups were largely distinct (Table S1). All these suggested that the five probiotics could result in alterations of gut microbiotas in both compositions and structures.

### Vital members in the gut microbiotas altered by LI09 or LI10

Some phylotypes associated with health or LD may contribute to the protective effects of both LI09 and LI10. One phylotype associated with health, i.e., OTU51_*Barnesiella*, was more abundant in both LI09 and LI10 groups than PC group, while OTU99_*Barnesiella* (associated with LD) was less abundant in both LI09 and LI10 groups than PC group, suggesting the two phylotypes could be associated with the protective effects of both LI09 and LI10.

All the five probiotics groups were included and analysed in this study, as the differences between the effective LI09/LI10 and the three other ineffective strains could help determine the vital bacteria in the gut microbiotas altered by LI09 or LI10. LEfSe results revealed that three representative OTUs (OTUs 51, 56 and 133) in LI09 group and two representative OTUs (OTUs 44 and 88) in LI10 group were assigned to *Barnesiella*, while two other representative OTUs (OTUs 12 and 285) in LI10 group were assigned to *Bacteroides*. *Barnesiella* and *Bacteroides* were enriched in the gut of rats receiving antibiotics against acetaminophen-induced acute LD at night (8 PM, light off) compared with daytime (8 AM, light on) [7], suggesting the representative phylotypes *Barnesiella* and *Bacteroides* to LI09 and/or LI10 groups may benefit more from the rest/night time.

OTU173_*Porphyromonadaceae* was determined with many correlations in the bacterial networks of both LI09 and NC groups, suggesting that OTU173_*Porphyromonadaceae* could help maintain the normal gut network in LI09 group. One representative OTU to LI09 group, i.e., OTU170_*Porphyromonadaceae*, was also determined as a gatekeeper to the bacterial network of LI09 group, suggesting OTU170_*Porphyromonadaceae* could play a vital role in the bacterial network of altered gut microbiota by LI09. *Porphyromonadaceae* was less abundant in the gut of rats induced with alcoholic liver disease (which represents a chronic wide-spectrum of LD) than healthy ones [20], suggesting *Porphyromonadaceae* plays a beneficial role in the gut microbiota altered by LI09 in the current study.

The functional metabolites could be associated with the protective effects of LI09 and LI10. K01278 - dipeptidyl peptidase 4 was most associated with LI09-treated microbiota. Dipeptidyl peptidase 4 plays a vital role in the development of liver diseases, i.e., non-alcoholic fatty liver disease, hepatic steatosis and hepatocellular carcinoma [21–23]. As LI09 was effective to alleviate LD in rats [15], the representative dipeptidyl peptidase 4 could be either as the functional metabolite that LI09 unable to inhibit, or as a side-effect of LI09 treatment. K00527 - ribonucleoside triphosphate reductase was most associated with LI10 treated microbiota. Increased levels of ribonucleoside triphosphate reductase genes were associated with the added Zn^2+^ to *Streptococcus pneumoniae* culture [24], suggesting the representative ribonucleoside triphosphate reductase to LI10 group could be associated with the increase of metal ion.

### Comparisons of the three clustered microbiotas

In the current study, the gut microbiotas in LI09, LI10 and PC groups were clustered into three clusters for subsequent analyses to determine the characteristics of LI09 or LI10 treated LD rats which were at different gut microbial colonization states. LDDR was highest in Cluster_2_Microbiota, and lowest in Cluster_3_Microbiota, suggesting Cluster_2_Microbiota was at least dysbiotic status, while Cluster_3_Microbiota was at most dysbiotic status. The liver function variables and plasma cytokines were lowest within Cluster_2 and relatively highest in Cluster_3. As liver function variables and plasma cytokines were lower in healthy group than LD groups [15], the relevant results about the variables in the current study suggested that Cluster_2 cohort were at best health status, with Cluster_3 cohort at the relatively worst health status among the three clustered LD cohorts.

Multiple associations between representative OTUs and liver function variables/plasma cytokines were found in Cluster_2, but not in Cluster_1 or Cluster_3. *Lachnospiraceae* was enriched in the gut of patients with less severe liver disease (i.e., nonalcoholic fatty liver disease) but decreased in those with more severe diseases (i.e., nonalcoholic steatohepatitis and liver cirrhosis) [25]. In Cluster_2 of the current study (which was determined at least dysbiotic status), OTU180_*Lachnospiraceae* had relatively complex correlations with five other representative OTUs and four variables (Fig. 6), suggesting OTU180_*Lachnospiraceae* and the five other OTUs could be associated with the changes of the host responses to benefit the protective effects of LI09 or LI10.

## Conclusion

Two groups of OTUs were associated with LI09 and LI10 groups, among which, OTU170_*Porphyromonadaceae* and OTU12_*Bacteroides* were vital to the gut microbiotas altered by LI09 and LI10, respectively. The characteristics of the LD cohorts treated by LI09 or LI10 at different gut microbial colonization states could help monitor the health status of LD cohorts treated by LI09 or LI10.

## Methods

### Probiotics and animal experiment

The preparation of five probiotic bacteria and the procedures for the animal experiments as described by in our previous study (Figure S4) [15]. *Bifidobacterium longum* LI06 (CGMCC 10385), *B. longum* LI07 (CGMCC 10386), *B. pseudocatenulatum* LI08 (CGMCC 10387), *B. pseudocatenulatum* LI09 (CGMCC 10388), and *B. catenulatum* LI10 (CGMCC 10389) were streaked onto trypticase phytone yeast agar plates and cultured anaerobically at 37 °C for 36 h, before being prepared at a final concentration of 3 × 10^9^ colony-forming units per ml in physiological saline. Male pathogen-free Sprague-Dawley rats weighting 250 to 350 g from the Experimental Animal Center of Zhejiang Province in China were included for this study. Rats were individually caged at 22 °C and exposed to a 12:12 light/dark cycle. A standard laboratory rat chow diet and free access to tap water were provided. All procedures were performed according to the 2011 National Institutes of Health Guide for the care and use of laboratory animals.

Fifty-nine rats were randomly allocated for seven groups, including five probiotics groups (*n* = 9 per group), one PC group (*n* = 8, with an intraperitoneal injection of D-galactosamine but no probiotics administration) and one NC group (*n* = 6, without an intraperitoneal injection of D-galactosamine or probiotics administration). Each experimental group was treated being orally administrated by each of the five probiotics for 7 days. Intraperitoneal injection of D-galactosamine was used to induce LD to the rats in all groups except NC group at a dose of 1.1 g/kg body weight on the eighth day, with much less hepatic pathological changes in LI09 and LI10 groups compared with PC group (Figure S5) [15]. The animals were anaesthetized with an intraperitoneal injection of 80 mg/kg ketamine and 10 mg/kg xylazine 24 h after the induction of LD, before being subjected to laparotomy through a midline incision and an eventual unconscious death. The blood and caecal content samples were collected for subsequent analyses. The protocols of the current study were approved by Animal Care and Use Committee of the First Affiliated Hospital, School of Medicine, Zhejiang University.

### Measurement of liver function variables

Multiple liver function variables, i.e., the serum levels of ALT, AST, GGT, GPDA, TBA, TB and ALB were measured by Fang et al. [15]. The dataset was used for achieving different objective in the present study.

### Plasma cytokines analysis

A series of plasma cytokines, specifically IL-1β, IL-5, IL-6, IL-10, monocyte chemoattractant protein 1 (MCP-1), macrophage colony-stimulating factor (M-CSF), macrophage inflammatory protein 1 alpha (MIP-1α), MIP-3α and tumour necrosis factor alpha (TNF-α) were measured by Fang et al. [15]. The dataset was used for achieving different objective in the present study.

### Molecular methods

DNA extraction from caecal contents and the following polymerase chain reaction (PCR) amplification were conducted by Fang et al. [15]. The 16S rRNA V3-V4 regions were targeted for DNA amplification using the fusion dual barcoded primers 319F/806R. The PCR products were purified before being sequenced on the Illumina MiSeq Instrument (Illumina Inc., USA) using 2 × 300 base pair paired-end protocol.

### Processing of sequencing data

The raw sequencing data were processed as described by Fang et al. [15]. DNA paired-end reads were merged and quality filtered using Usearch sequence analysis tool. Chimera filtering occurred prior to clustering of OTUs based on identity threshold at ≥97% against Ribosomal Database Project (RDP) database.

### Comparisons of the gut microbiotas in the seven groups

The gut microbiotas in the seven groups were compared to investigate the vital bacteria to the gut microbiotas altered by LI09 or LI10. PERMANOVA was performed in R software version 3.5.2 using the vegan package [26], to compare the gut compositions between the seven groups. SIMPER analysis was used to identify the dissimilarities in the gut compositions between LI06 - LI10 groups and PC group, as well as the dissimilarities in the gut compositions between LI06 - LI10 groups and NC group. The dissimilarities of gut compositions between the five probiotics groups and PC group were compared with those between five probiotics groups and NC group by using a t-test.

### OTUs and functional metabolites associated with the gut microbiotas altered by LI09 or LI10

A LEfSe analysis was used to determine the representative OTUs (i.e., OTUs capable of differentiating microbiota in one group from those of the six other groups) to each of the seven groups. The OTUs with LDA threshold ≥3.0 and consistently significant across each of the seven groups were identified as representative OTUs to the corresponding groups.

The functional metabolites for the seven groups were determined by using Tax4fun package based in R software [27]. A LEfSe analysis was carried out to determine the functional metabolites associated with the gut microbiotas of the seven groups.

### Microbiological networks and fragmentation analyses

CoNet analysis was used to determine the correlations of the OTUs in each of the seven gut microbiotas, based on an ensemble of correlation measures as described by Faust et al. [28]. The top 10 OTUs with most correlations in each bacterial network of the seven groups were determined.

Network fragmentation calculations and generation of a null distribution were carried out in R using the package igraph [29], 1) to determine whether any representative OTU or top 10 OTUs with most correlations in the seven groups was also as gatekeepers to the corresponding microbiota, and 2) to investigate the gatekeepers in each of bacterial networks of the seven groups. The details of this analysis were described by Wagner Mackenzie et al. [30].

### Changes of OTUs associated with LD and health in LI09 and LI10 groups

A LEfSe analysis was used to determine the OTUs differentiating the microbiotas between PC and NC groups. The OTUs with LDA threshold ≥3.0 and consistently significant across the PC or NC groups were identified as OTUs associated with LD or health. A Kruskal-Wallis test was performed to compare the abundances of OTUs associated with LD between LI09, LI10 and PC groups, with Mann-Whitney test for pairwise comparisons. The same techniques were applied to compare the abundances of OTUs associated with health between LI09, LI10 and PC groups.

### Clustering of gut microbiotas from LI09, LI10 and PC groups

The gut microbiotas from LI09, LI10 and PC groups were clustered by PAM clustering analysis based on their bacterial compositions, with each cluster owning varied numbers of microbiotas. Before PAM clustering, the average silhouette method was used to determine the optimal numbers of clusters for the three groups of gut microbiotas [19].

Three clustered cohorts at three gut microbial colonization states were determined for the subsequent analyses, to investigate the characteristics of the LD rats treated by LI09 or LI10 at different gut microbial colonization states.

### Comparisons of the gut microbiotas of the three clustered cohorts

One-way ANOVA was performed to compare the alpha diversity (i.e., richness, diversity and evenness) between the three clustered microbiotas, followed by t-tests for pairwise comparisons. PERMANOVA was applied to compare the gut compositions of the three clustered microbiotas.

Dysbiosis ratios of bacterial taxa are associated with different diseases [31–33]. For example, cirrhosis dysbiosis ratio, i.e., the abundance ratio of “good and bad” gut taxa, was associated with the severity of liver cirrhosis [34]. In the current study, LDDR was defined as the abundance ratio of health associated OTUs and LD associated OTUs, to help determine the dysbiosis statuses of the three clustered gut microbiotas. LDDRs of the three clustered microbiotas were transformed in log10 to satisfy the assumptions of normal distribution and equal variance, before being compared by one-way ANOVA, with t-tests for the pairwise comparisons.

### Comparisons of liver function variables and plasma cytokines in the three clustered cohorts

One-way ANOVA was applied for the comparisons of liver function variables ALB, ALT, AST, GGT and GPDA among the three clustered cohorts originally from LI09, LI10 and PC groups, as well as the plasma cytokines M-CSF and TNF-α, followed by t-tests for pairwise comparisons.

TBA, TB, IL-1β and IL-6 of the three clustered cohorts were transformed in log10, and IL-5, IL-10, MIP-1α, MIP-3α and MCP-1 were transformed in square root, to satisfy the assumptions of normal distribution and equal variance. One-way ANOVA was used to compare the transformed liver function variables and plasma cytokines, with t-tests for the pairwise comparisons.

The *P* values in all the pairwise comparisons in the current study were adjusted by Bonferroni correction. The statistical analyses were performed using SPSS Statistics software version 25 (IBM Inc., USA).

### Associations of representative OTUs in the three clustered microbiotas with the liver function variables and plasma cytokines

A LEfSe analysis was performed to identify the representative OTUs for each of the three clustered microbiotas. CoNet analysis was applied to determine the associations of the representative OTUs with the liver function variables and plasma cytokines in each of the three clustered cohorts.

## Supplementary information

**Additional file 1:Table S1.** Ten OTUs with most correlations in each bacterial network of the seven groups identified by co-occurrence network inference analysis.

**Additional file 2:Table S2.** Gatekeepers in the gut microbiota of each of the seven groups determined by fragmentation analysis.

**Additional file 3:Table S3.** Gut microbiotas in LI09, LI10 and PC groups clustered into three different clusters by PAM clustering analysis.

**Additional file 4:Table S4.** Comparisons of richness, diversity and evenness indices among the three clustered gut microbiotas.

**Additional file 5:Figure S1.** Co-occurrence network inference analysis determines the bacterial correlations in each of the seven groups.

**Additional file 6:Figure S2.** OTUs associated with health (i.e., NC) and disease (i.e., PC) identified by LEfSe.

**Additional file 7:Figure S3.** Optimal cluster numbers with corresponding scores identified by silhouette analysis.

**Additional file 8:Figure S4.** Brief schematic diagram of the experimental design.

**Additional file 9:Figure S5.** Hepatic pathology of the rats in the seven groups.

## Data Availability

All the data generated or analysed in the present study are available from the corresponding author upon reasonable request.

## Abbreviations

SIMPER: Similarity percentage
PERMANOVA: Permutation analysis of variance
PAM: Partition Around Medoids
CoNet: Co-occurrence Network inference
ALT: Alanine aminotransferase
AST: Aspartate aminotransferase
GGT: Glutamyltransferase
GPDA: Glycylproline dipeptidyl aminopeptidase
TBA: Total bile acid
TB: Total bilirubin
ALB: Albumin
IL: Interleukin
MCP-1: Monocyte chemoattractant protein 1
M-CSF: Macrophage colony-stimulating factor
MIP-1α: Macrophage inflammatory protein 1 alpha
TNF-α: Tumour necrosis factor alpha
